# Supplementary material for: YM155 Inhibition of Survivin Enhances Carboplatin Efficacy in Metastatic Castration-Resistant Prostate Cancer
Source: Pharmaceuticals (Basel). 2025 Nov 18;18(11):1752. doi: 10.3390/ph18111752 (PMC12655369; doi:10.3390/ph18111752)
Supplement: Supplementary file 1 [file pharmaceuticals-18-01752-s001.zip › pharmaceuticals-3995795-supplementary.pdf]

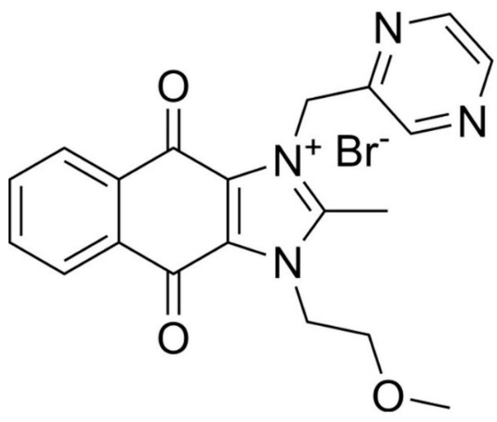

Supplementary Figure S1. YM155 (Sepantronium bromide) chemical structure

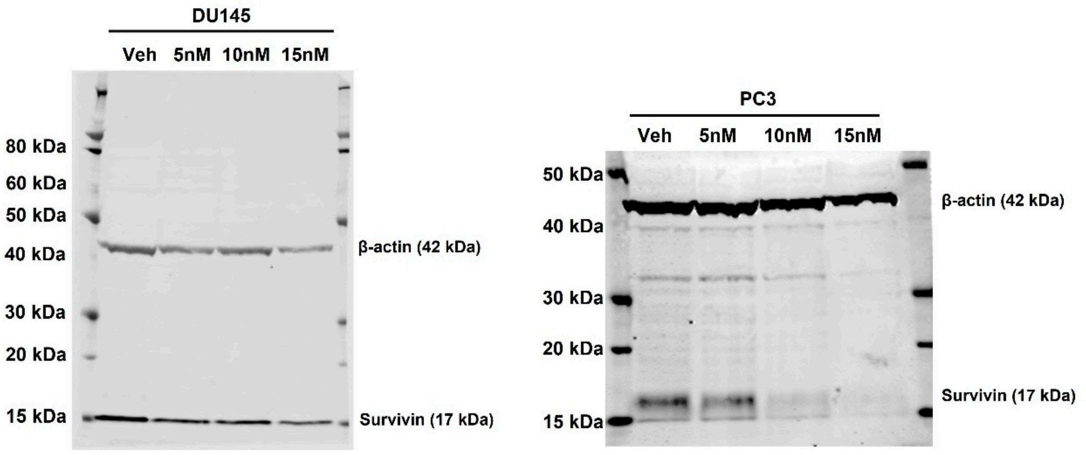

Supplementary Figure S2. Uncropped western blot images
